# Building interpretable fuzzy models for high dimensional data analysis in cancer diagnosis

**DOI:** 10.1186/1471-2164-12-S2-S5

**Published:** 2011-07-27

**Authors:** Zhenyu Wang, Vasile Palade

**Affiliations:** 1 Computing Laboratory, Oxford University, Oxford, OX1 3QD, UK

## Abstract

**Background:**

Analysing gene expression data from microarray technologies is a very important task in biology and medicine, and particularly in cancer diagnosis. Different from most other popular methods in high dimensional bio-medical data analysis, such as microarray gene expression or proteomics mass spectroscopy data analysis, fuzzy rule-based models can not only provide good classification results, but also easily be explained and interpreted in human understandable terms, by using fuzzy rules. However, the advantages offered by fuzzy-based techniques in microarray data analysis have not yet been fully explored in the literature. Although some recently developed fuzzy-based modeling approaches can provide satisfactory classification results, the rule bases generated by most of the reported fuzzy models for gene expression data are still too large to be easily comprehensible.

**Results:**

In this paper, we develop some Multi-Objective Evolutionary Algorithms based Interpretable Fuzzy (MOEAIF) methods for analysing high dimensional bio-medical data sets, such as microarray gene expression data and proteomics mass spectroscopy data. We mainly focus on evaluating our proposed models on microarray gene expression cancer data sets, i.e., the lung cancer data set and the colon cancer data set, but we extend our investigations to other type of cancer data set, such as the ovarian cancer data set. The experimental studies have shown that relatively simple and small fuzzy rule bases, with satisfactory classification performance, can be successfully obtained for challenging microarray gene expression datasets.

**Conclusions:**

We believe that fuzzy-based techniques, and in particular the methods proposed in this paper, can be very useful tools in dealing with high dimensional cancer data. We also argue that the potential of applying fuzzy-based techniques to microarray data analysis need to be further explored.

## Background

Microarray techniques allow simultaneous measuring of the expression of thousands of genes under different experimental environments and conditions. They allow us to analyse the gene information very rapidly by managing them at one time. The gene expression profiles from particular microarray experiments have been widely used for cancer classification [1-3]. However, the amount of data produced by this new technology is usually too large to be manually analysed. Hence, the need to automatically analyse the microarray data offers an opportunity for Machine Learning (ML) methods to have a significant impact on cancer research.

The data from a series of *m* microarray experiments can be represented as an *m* × *n* gene expression matrix (see Table 1), where each row represents a sample described by the expression of *n* genes from one experiment. Each sample belongs to a certain class, i.e., cancer or non-cancer. Compared to some other classical problems in machine learning, microarray data sets pose various problems. The number of features (genes), usually in the range of 2,000-30,000, is much larger than the number of examples (usually in the range of 40-200). In addition, microarray data often brings in multiple missing gene expression values and noisy signals from the experiments. Therefore, classifying cancer mi-croarray gene expression data can be regarded as a high-dimensional-low-sample data problem with lots of noisy or missing data.

**Table 1:** A typical gene expression matrix *X*, where rows represent samples obtained under different experimental conditions and columns represent genes

	Gene 1	Gene 2	…	Gene n-1	Gene n	Class
1	165.1	276.4	…	636.6	784.9	1

2	653.6	1735.1	…	524.1	104.5	-1

…	…	…	…	…	…	…

m-1	675.0	45.1	…	841.9	782.8	-1

m	78.2	893.8	…	467.9	330.1	1

Unsupervised methods, such as Clustering [4], and Self-Organizing Maps (SOMs) [5] were initially used to analyse the relationships among different genes. Subsequently, supervised methods, such as Support Vector Machines (SVMs) [6], Multi-Layer Perceptrons (MLPs or NNs) [7,8], K Nearest Neighbor (KNN) methods [9,10], etc., have been successfully applied to the classification of different tissues. But, most of the current methods in microarray data analysis are black box methods; these models can not satisfactorily reveal the hidden information in the data. This information usually plays a very important role in making a quality clinical diagnosis.

Different from black-box methods, fuzzy rule-based models can not only provide good classification results, but also easily be explained and interpreted in human understandable terms by using fuzzy rules. This provides the researchers or clinician an insight into the developed models. At the same time, fuzzy systems adapt numerical data (input/output pairs) onto human linguistic terms and offer very good capabilities of dealing with noisy and missing data. Compared to other popular rule-based models in the area, such as C4.5 Decision Trees (DTs) [11,12], the linguistic rules generated by our fuzzy-based models are short and easy to be read.

Unfortunately, rule-based methods have suffered some well-known limitations in dealing with high dimensional data. Very high dimensional feature vectors and lack of enough training samples are two major challenges for modeling microarray data in general, hence for the success of applying fuzzy models to this problem too. However, some recent developments in fuzzy systems provide us with some good ways to obtain good diagnosis results. For example, Vinterbo et al. [13] firstly used fuzzy rule bases to classify gene expression data, but this model only allow linear discrimination, and the classification performance is limited; an Adaptive-Network-based Fuzzy Inference System (ANFIS) was successfully applied for this problem in [14] too; Woolf and Wang [15] developed a fuzzy based model to analyse the relationships between genes, while Ressom et al. [16] used a clustering-based preprocessing method to increase the efficiency of the fuzzy models. All these reported systems are either small models which perform well on small data sets, or huge models which are difficult to be understood by the human experts. Other machine learning techniques, like genetic algorithms (GA) [17] or ensemble learning [18,19] have been adopted to allow fuzzy rule-based models to deal with a relative large number of features, but the obtained rule bases still look very complex to be easily comprehensible. Large model complexity significantly damages the main advantage of applying fuzzy models to this application, i.e., the inter-pretability of the models. The computational cost of constructing these models is generally very high too.

Normally, the accuracy of each fuzzy rule-based classifier is measured by the number of correctly classified training or testing patterns, while its in-terpretability is measured by the complexity of the model, more specifically, the number of fuzzy rules and the total number of antecedent conditions. Whereas both accuracy and interpretability were considered, multi-objective evolutionary based methods are introduced into our systems, hence, the name of Multi-Objective Evolutionary Algorithms based Interpretable Fuzzy (MOEAIF) models. We evaluated our proposed model on some well-known cancer data sets, i.e., the ovarian cancer data set, the lung cancer data set and the colon cancer data set. Experimental results are listed and discussed in the later section. Compared with most previously reported models, accurate and small fuzzy rule bases were obtained.

## Methods

### Gene Selection

A major goal for diagnostic research is to develop diagnostic procedures based on inexpensive microarrays that have enough probes to detect certain diseases. This requires the selection of some genes which are highly related to the particular classification problem, i.e., the informative genes. This process is called Gene Selection (GS), which corresponds to feature selection from any machine learning task in general. Two basic approaches for feature selection used in machine learning and information theory literature are the filter methods and the wrapper methods [9,20,20,21]. In theory, wrapper methods should provide more accurate classification results than filter methods [21]. The main disadvantage of the wrapper approach is its computational cost when combined with more complex algorithms such as SVM for example. The wrapper approach, which is popular in many machine learning applications, is not extensively used in DNA microarray tasks, and in most cases the gene selection is performed by ranking genes on the basis of scores, correlation coefficients, mutual information and sensitivity analysis. More detailed discussions of these two approaches can be found in [9,20,22-24]. As suggested in [25], a Fuzzy C-Mean Clustering based Enhanced Gene Selection method (FCCEGS) is applied in this paper as well for gene selection.

### Improved Methods for Obtaining Interpretable Fuzzy Models

An ideal design of fuzzy rule-based models for microarray data analysis is when we find fuzzy rule-based models with good interpretability but with acceptable testing accuracy too. Compared to most popular methods in cancer microarray gene expression data analysis area, rule-based fuzzy models usually have relative high computational complexity. In order to obtain good fuzzy rule-based models, we adopted the following recent techniques to reduce the complexity of fuzzy rule-based models.

#### Weighted Fuzzy Rules

The fuzzy rules *R_q_* used in our models are in the form of:

• *R_q_*: If *x*_1_ is *A_q_*_1_ and … and *x_n_* is *A_qn_*, then Class *C_q_* with *CF_q_.*

In the above rule, *x* = (*x*_1_,…,*x_n_*) is the n-dimensional input vector, *A_qi_* is an antecedent fuzzy set for the *i – th* input variable, *C_q_* is a consequent class, and *CF_q_* is a certainty degree (i.e., rule weight). The rule weight is a real number in the unit interval [0, 1].

#### Multiple Fuzzy Partitions

**Figure 1:**
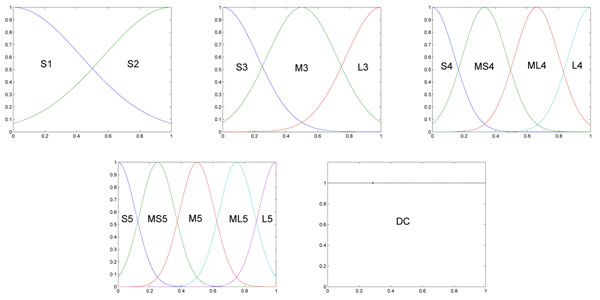
**Membership functions from multiple fuzzy partitions.** 15 membership functions from four fuzzy partitions of the domain interval [0, 1]. S, MS, M, ML and L denote Small, Medium Small (relatively small), Medium, Medium Large (relatively large) and Large, respectively. DC denotes “Don’t Care” membership function.

For a high-dimensional problem like microarray data analysis, the antecedent conditions of the generated rules are normally very numerous. Short fuzzy if-then rules with only a few antecedent conditions are obviously easy to understand for human users, and therefore a novel technique has been applied, as explained below. We simultaneously generated 14 fuzzy sets from multiple fuzzy partitions, as shown in Figure 1; “S”, “MS”, “M”, “ML” and “L” denote Small, Medium Small (relatively small), Medium, Medium Large (relatively large) and Large, respectively. The “don’t care” (DC) condition has been added as an additional set. There are 15 new fuzzy sets in total, and all of these fuzzy sets are fixed, without any tuning mechanism during the training. After training, some fuzzy if-then rules may have *n* antecedent conditions (i.e., have no DC conditions), and others may have only a few antecedent conditions (i.e., have more DC conditions).

#### Simple Fuzzy Reasoning

Since we have 15 antecedent fuzzy sets for each attribute of our n-dimensional pattern classification problem, the total number of combinations of the antecedent fuzzy sets is 15*^n^*. Each combination is used as the antecedent part *A_q_* of the fuzzy rule *R_q_.* Its consequent class *C_q_* and rule weight *CF_q_*are specified from compatible training patterns with *A_q_* in the following heuristic manner.

First, we calculate the compatibility degree of each pattern *x_p_* with the antecedent part *A_q_* of the rule *R_q_* via a product operation like:

*µ_A_q__*(*x_p_*) = *µ*_A_q1__(*x_p_*_1_) ⋅…⋅*µ_A_qn__*(*x_pn_*) (1)

where *µ_A_q__*(•) is the membership function of *A_qi_.* Then, the confidence of the fuzzy rule *A_q_* ⇒ *Class h* is calculated for each class *h* as follows:(2)

where *m* denotes the number of training patterns.

The consequent class *C_q_* is specified by identifying the class with the maximum confidence:(3)

where *M* is the number of classes.

When there is no pattern in the fuzzy sub-space defined by *A_q_*, we do not generate any fuzzy rules with *A_q_* in the antecedent part. This specification method of the consequent class of fuzzy rules has been used in a few studies since early 1990s [26]. It should be noted that the same consequent class as in Equations 2 and 3 is obtained when we use the support of the fuzzy rule *A_q_* ⇒ *Class h* instead of the confidence degree. The support of the fuzzy rule is calculated as follows:(4)

Different specifications for the rule weight *CF_q_* have been proposed and examined. In this paper, we only consider binary classification problem, we can use the following specification because good results were previously reported for that in these papers [27,28]:(5)

Let *S* be a subset of candidate fuzzy rules, i.e., a fuzzy rule-based classifier. Each pattern *x_p_* is classified by a single winner rule *R_w_*, which is chosen from the rule set S as follows:

(6) *µ_A_w__*(*x_p_*) ⋅ *CF_w_* = *max*{*µ_A_q__*(*x_p_*) ⋅ *CF_q_*|*R_q_* ∈ *S*}.

We only generate short fuzzy rules with a few antecedent conditions, and it should be noted that the DC conditions can be omitted from fuzzy rules. This restriction is used in order to find a compact set of fuzzy rules with high interpretability. As for short fuzzy rules, we only use fuzzy rules that satisfy both the minimum confidence and support as candidate rules for the multi-objective genetic fuzzy rule selection mechanism.

Rule confidence and support can be used as pre-screening criteria for finding a tractable number of candidate fuzzy rules. The generated fuzzy if-then rules are then divided into *T* groups according to their consequent classes, where *T* is a user-defined parameter. Fuzzy if-then rules in each group are sorted in the descending order of a pre-screening criterion (i.e. confidence, support, or their product). For selecting *Q* candidate rules, the first *Q/T* rules are chosen from each of the *T* groups, and in this manner, we can choose an arbitrarily specified number of candidate fuzzy if-then rules (i.e., *Q* candidate rules). It should be noted that the aim of the candidate rule pre-screening is not to construct a fuzzy rule-based system, but to find candidate rules, from which a small number of fuzzy if-then rules are later selected. For using a variety of candidate rules in rule selection, we choose the same number of fuzzy if-then rules (i.e., *Q/T* candidate rules) for each class. By applying these new techniques, the models complexity and computational cost is significantly decreased.

### The Multi-Objective Evolutionary Algorithms based Interpretable Fuzzy (MOEAIF) Model

In this section, we describe how to apply multi-objective evolutionary algorithms (MOEA) to extract fuzzy rule sets considering the balance between model accuracy and model interpretability.

Our task is to select a smaller number of simple fuzzy if-then rules with high classification performance, and this is performed by maximizing the classification accuracy, minimizing the number of selected rules, and minimizing the total rule length at the same time. Therefore, the fitness value of each string *S* (i.e., each rule set *S*) in the current population is defined by the three objectives using the following fitness function:

(7) *f*(*S*) = *w*_1_⋅*NCCP*(*S*) *–w*_2_⋅*NOR*(*S*) *–w*_3_⋅*NOA*(*S*),

where *w* = (*w*_1_, *w*_2_,*w*_3_), *NCCP*(*S*), *NOR*(*S*), and *NOA*(*S*) are the weight vector, the number of correctly classified training patterns, the number of selected fuzzy rules in *S*, and the total number of antecedent conditions in *S*, respectively. The weights w_1_,*w*_2_,*w*_3_ must satisfy the following conditions:

(8) *w*_1_,*w*_2_,*w*_3_≥0;

(9) *w*_1_+*w*_2_+*w*_3_ = 1;

As suggested by [29,30], a rule subset *R* consisting of the *Q* candidate rules can be represented by a binary string as:

(10) *R* = *r*_1_*r*_2_*r*_3_…*r_Q_*,

where *r_q_* = 0 means that the *q*-th candidate rule *r_q_* is not included in the rule set *R*, while *r_q'_* = 1 means that *r_q'_* is included in *R*.

To keep the model complexity low, a simple two-points crossover strategy is applied to each pair of parent strings to generate a new string. Biased mutation is applied to the generated string to efficiently decrease the number of fuzzy if-then rules included in each string, that is, different mutation probabilities are used for the mutation from 1 to 0 and the one from 0 to 1. For example, the mutation probability from 1 to 0, , and that from 0 to 1, , are defined as:(11)(12)

where *w*_1_,*w*_2_,*w*_3_ are the user defined weights of the three search objectives in the fitness function, and *PA*_10_ and *PA*_10_ are two initial fixed parameters. A larger probability is normally assigned to the mutation from 1 to 0 than to that from 0 to 1, in order to efficiently decrease the number of fuzzy if-then rules (i.e., the number of 1s) included in each string. By applying the Equations 11 and 12, the mutation rate can be automatically adjusted according to user different purposes. Our MOEAIF can be summarized as follows:

1. *Step 1:* Randomly generate *N_pop_* (number of individuals in the population) binary strings of length *Q* as an initial population. Specify the crossover probability *p_c_*, two mutation probabilities, *PA*_10_ and *PA*_10_, and the stopping condition;

2. *Step 2:* Generate *N_pop_* children strings by applying crossover and mutation to the current population;

3. *Step 3:* Calculate the three-objectives fitness value for each string; unnecessary rules are removed from each string;

4. *Step 4:* Update the next population by selecting top ranked individuals;

5. *Step 5:* Stop, if the stopping condition is satisfied or the maximum number of training epochs is reached, otherwise return to Step 2.

## Results and Discussion

### Cancer Data Sets

We evaluated our proposed MOEAIF models on three cancer data sets, namely the ovarian cancer data set, the lung cancer data set and the colon cancer data set.

• **Lung Cancer Data Set**

Lung Cancer Classification differentiates between malignant pleural mesothe-lioma (MPM) and adenocarcinoma (ADCA) of the lung. There are 181 reported samples in total, where 31 samples belong to MPM and 150 samples belong to ADCA. The training set contains 32 samples, 16 MPM and 16 ADCA, and the remaining 149 samples are used for testing. The expression levels of 12,533 features were report in each sample. Each feature represents one probe, for example, the feature 1018.at represents the probe 1018 at. The data is available at http://cilab.ujn.edu.cn/datasets.htm.

• **Ovarian Cancer Data Set**

The ovarian cancer data set was first reported in [31]. The aim of the experiment was to identify proteomic patterns in serum that distinguish ovarian cancer from non-cancer. This study is significant to women who have a high risk of ovarian cancer due to family or personal history of cancer. The proteomic spectra were generated by mass spectroscopy and the raw data can be found at http://clinicalproteomics.steem.com. There are 253 reported samples in this data set, where 91 samples belong to normal and 162 samples belong to ovarian cancers. The normalization is done over all the 253 samples for all 15154 M/Z identities. After the normalization, each intensity value is to fall within the range of 0 to 1. The data is available at http://cilab.ujn.edu.cn/datasets.htm.

• **Colon Cancer Data Set**

The data set used here was firstly reported in [1]. This data set contains 62 samples, of which 40 are tumour samples, and 22 normal samples. About 6000 genes are represented in each sample in the original data set, out of which only 2000 were selected. The data is available at http://sdmc.i2r.astar.edu.sg/rp/ColonTumor/ColonTumor.html.

### Accuracy of the MOEAIF Models

Different sets of *w*_1_, *w*_2_ and *w*_3_ are used to simulate different users’ requirements. From Table 2 and Table 3, we can see that when classification accuracy is preferred, we can achieve the highest testing accuracy, at 91.28% on lung cancer data, and 86.71% on ovarian cancer data. When the interpretability of the models is preferred, we can use two rules to classify lung cancer data with 89.26% testing accuracy, and three rules to classify lung cancer data with a testing accuracy of 91.28%. Very small rule bases for the lung cancer data set are obtained. Due to lack of enough training examples, a satisfactory testing accuracy on the colon data set was not obtained.

**Table 2:** Classification accuracy and interpretability of models on the lung cancer data set.

*w*_1_	*w*_2_	*w*_3_	Number of Rules	Average Rule Length	Testing Accuracy
0.1	0.7	0.2	2	1.5	89.26
0.5	0.1	0.4	6	1.8	90.06
0.5	0.4	0.1	3	2	90.06
0.5	0.2	0.2	3	2	89.93
0.7	0.1	0.2	3	2	91.28
1	0	0	23	2	90.06

**Table 3:** Classification accuracy and interpretability of models on the ovarian cancer data set.

*w*_1_	*w*_2_	*w*_3_	Number of Rules	Average Rule Length	Testing Accuracy
0.7	0.2	0.1	36	2.3	86.71
0.5	0.2	0.3	16	2	78.03
0.3	0.4	0.3	8	2	63.75

### Interpretability of the MOEAIF Models

We further explore the rule base obtained by our MOEAIF approach for lung cancer data set, where *w*_1_=0.5, *w*_2_=0.3, and *w*_3_=0.2.

**Figure 2:**
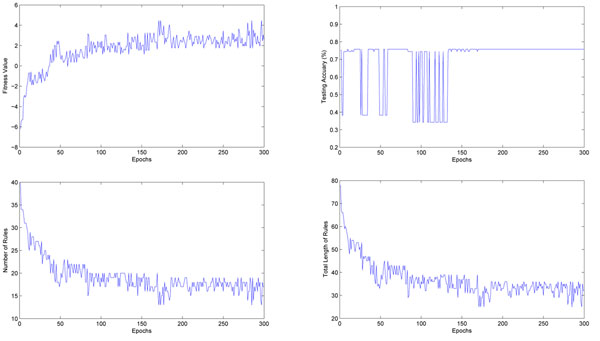
**The rule extraction process for the lung cancer data set.** Left (UP): The fitness value of the best rule base found in the population; Right (UP): The testing accuracy given by the best rule base; Left (Down): The total number of fuzzy rules in the rule base; Right (Down): The sum of the length of all rules in the rule base.

The whole training process is given in Figure 2. The up left figure shows the fitness value of the best rule base found in the population during the training, the up right figure shows the testing accuracy given by the best rule base, the total number of fuzzy rules in the rule base is given in the lower left figure, and the total length of rules in the rule base is given in the lower right figure. From these figures, a wide fluctuation of testing accuracy (right figure (up)) frequently appears at the early stage of training. By analyzing the population, we notice this phenomenon means there must exist some very strong fuzzy rules which have large effect on the final classification result. Including these rules in the selected rule subset will significantly change the fitness value and classification accuracy during training. In the training stage, this phenomenon can suggest to the users that a small, but useful fuzzy rule set exists in this data set. A good testing accuracy is obtained within the first few generations, which shows that the pre-screening of candidate rules plays a very important role in this case. Multiple fuzzy partition technique also helps us avoid the high computational cost of adjusting fuzzy membership functions like some previously built fuzzy models.

**Figure 3:**
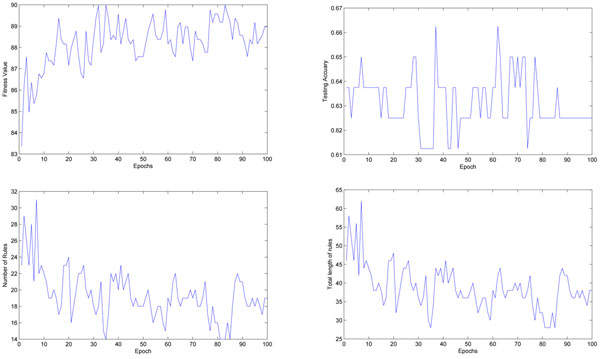
**The rule extraction process for the ovarian cancer data set.** Left (UP): The fitness value of the best rule base found in the population; Right (UP): The testing accuracy given by the best rule base; Left (Down): The total number of fuzzy rules in the rule base; Right (Down): The sum of the length of all rules in the rule base.

Compared to the lung cancer data set, it is more difficult to find an efficient small rule subset for the ovarian cancer data, see Figure 3, where the name of features are short for the names of M/Z identities. Ovarian cancer data normally require a large number of genes, a large rule subset and a large number of initial candidate rules to obtain acceptable testing accuracy. The algorithm can not converge when the number of the input genes is smaller than 8. Because the pre-processing stage has already defined a small search space, there is no need to set the number of generations to be a large number. It would not help the algorithm to converge if some useful rules are not in the initial rule base, and the testing accuracy can also be difficult to be further improved during the training.

### Rule Extraction

We can easily extract fuzzy rules from the lung cancer data set by using a trained MOEAIF model. This fuzzy rule base can classify the testing data with the accuracy of 0.8993 by using only three rules (see Tables 4). Four input features are used in the model, i.e., the feature 40256.at, the feature 1018.at, the feature 35792.at and the feature 33357.at. From this table, we can see that the rules obtained by our MOEAIF model are linguistically interpretable, for example:

**Table 4:** The selected rule subset for lung cancer data when testing accuracy = 0.8993; “–” denotes “don’t care” condition.

	40256.at	1018.at	35792.at	33357.at	CF	Class
Rule 1		-	-		0.9999	1

Rule 2	-		-		0.9829	-1

Rule 3	-			-	0.9725	-1

• Rule 1: If the feature 40256.at is “large” and the feature 33357.at is “large”, then the sample is Cancer with CF=99.99%.

• Rule 2: If the feature 1018.at is “large” and the feature 33357.at is “medium”, then the sample belongs to Normal with CF = 98.29%.

• Rule 3: If the feature 1018.at is “large” and the feature 35792.at is “relatively small”, then the sample belongs to Normal with CF = 97.25%.

The membership functions of the feature 1018.at and the feature 35792.at in *Rule*1 are “dont’t care”, which can reduce the length of *Rule*1. The rules generated by our MOEAIF models are shorter than the rules from our previously built models [14,18,25] and some other reported rule-based models [11,32].

Table 5 gives a rule base generated for the ovarian cancer data set by using the MOEAIF approach. The rule bases generated from the ovarian cancer data set are normally larger than the rule bases generated from the lung cancer data set. There are 8 fuzzy rules in this rule base and the average length of the rules is 2. But this eight short rules can classify the testing data with an accuracy of 0.6375. Six features are used in the model, and the feature MZ6880.2 and the feature MZ18871.5 play important roles in most of the rules.

**Table 5:** The selected rule subset for ovarian cancer data when testing accuracy = 0.6375. “–” denotes “don’t care”.

	MZ820.8	MZ6880.2	MZ1730.9	MZ1866.7	MZ18871.5	MZ827.3	Class
Rule 1	-			-	-	-	1

Rule 2	-		-	-	-		1 (0.9995)

Rule 3	-		-	-		-	1 (0.9994)

Rule 4	-	-	-	-			-1 (0.9999)

Rule 5	-	-	-			-	-1 (0.9999)

Rule 6	-		-	-		-	-1 (0.9997)

Rule 7	-		-	-		-	-1 (0.9996)

Rule 8		-	-	-		-	-1 (0.9994)

## Conclusions

In this paper, small and linguistically understandable fuzzy rule bases were obtained from challenging high dimensional cancer data sets by using our proposed Multi-Objective Evolutionary Algorithms based Interpretable Fuzzy (MOEAIF) method. The classification performance obtained by our models is also competitive. We also point out that an ideal design of fuzzy rule-based models for microarray gene expression data analysis includes two important tasks: designing low-complexity fuzzy models, and finding trade-off points between classification accuracy and model interpretability. We believe that fuzzy techniques and, in particular, the methods proposed in this paper can be very useful tools in dealing with microarray data. There also are some important issues that need to be addressed in the future. For example, some microarray gene expression data sets were generated directly from the probes set, and in some cases several probes may correspond to the same gene, or several different genes may hybridise to the same probes (i.e.,cross-hybridisation). If some of the input features (or probes) in a single rule are specific to the same gene(s), then this rule need to be deleted. Due to lack of enough training examples, satisfactory classification results were not always guaranteed in some small data sets, for example, the colon cancer data set in this paper.

## Competing interests

The authors declare that they have no competing interests.

## Authors contributions

Zhenyu Wang has done the experiments and drafted the text, while Vasile Palade has contributed in guiding the experiments and checking and improving the text of the paper.
